# Quantifying Optical Microangiography Images Obtained from a Spectral Domain Optical Coherence Tomography System

**DOI:** 10.1155/2012/509783

**Published:** 2012-06-26

**Authors:** Roberto Reif, Jia Qin, Lin An, Zhongwei Zhi, Suzan Dziennis, Ruikang Wang

**Affiliations:** Department of Bioengineering, University of Washington, Seattle, WA 98195, USA

## Abstract

The blood vessel morphology is known to correlate with several diseases, such as cancer, and is important for describing several tissue physiological processes, like angiogenesis. Therefore, a quantitative method for characterizing the angiography obtained from medical images would have several clinical applications. Optical microangiography (OMAG) is a method for obtaining three-dimensional images of blood vessels within a volume of tissue. In this study we propose to quantify OMAG images obtained with a spectral domain optical coherence tomography system. A technique for determining three measureable parameters (the fractal dimension, the vessel length fraction, and the vessel area density) is proposed and validated. Finally, the repeatability for acquiring OMAG images is determined, and a new method for analyzing small areas from these images is proposed.

## 1. Introduction

The appearance of tissue vasculatures may be an important biomarker to distinguish healthy from diseased tissues in several medical applications. For example, a change in retinal vessels is an early indicator of coronary heart disease [1, 2] and stroke [3]. Vascular remodeling has also been of interest in several fields including wound healing [4], oncology [5], and tissue regeneration [6].

Numerous techniques have been applied to obtain the vascular morphology from biological tissues. Optical intrinsic signal imaging [7], laser speckle imaging [8], and laser-Doppler flowmetry techniques [9] can obtain microangiography images; however, they have low spatial resolution which limits their capability for viewing small capillaries. Confocal microscopy provides high spatial resolution; however, it is limited by its penetration depth and requires the use of fluorescent tissue markers [10]. Photoacoustic imaging, based on thermal-acoustic phenomena resulting from the strong light absorption of  blood and the subsequent thermo-elastic expansion, provides high-resolution angiography images; however, the acquisition time is slow [11, 12].

Optical microangiography (OMAG) is a method for obtaining three-dimensional images of blood vessels *in vivo*, using a spectral domain optical coherence tomography (OCT) system [13, 14]. OMAG is based on separating the static from dynamic tissue structures, which is done by detecting the changes in the scattered signal through time, due to the movement of particles such as red blood cells. OMAG has been used in several studies such as visualizing the corneal-sclera limbus [15], the retina [16], the skin [17] and the cerebral [18], and renal microcirculations [19]. OMAG has the advantage that it can capture a large image (2 × 2 mm) within a few seconds with high resolution (*∼*10 *μ*m) and high penetration depth (*∼*2.5 mm).

 Microangiography images provide direct visualization of the blood vessels and capillaries within biological tissues. Usually, these images are interpreted qualitatively [20]. Previous methods to quantify these images include measuring blood vessel diameters [21], the flow velocity [22], and the maximum distance in the tissue to the nearest blood vessel [23]. New methods that can enable the quantification of the blood vessels, such as the vessel tortuosity, will be beneficial for several studies, like angiogenesis.

 Vessel length fraction and vessel area density are parameters which represent a relative value of the total length of the vessels and the total area occupied by the vessels, respectively [24]. Fractal dimension (FD) is an approach that is used to characterize images [25]. A fractal dimension is a value that gives an indication of how an image fills space as one zooms into smaller scales. It has also been defined as a description of blood vessel tortuosity. Fractal analysis enables the assessment of the architecture of a vascular network, particularly the branching of vessels. It has been applied in diverse areas of medicine to describe complex biological structures [26] such as branching patterns of the retina [27], coronary [28], and pulmonary arterioles [29]. It has also been used to quantify the fractal distribution of scatterers in tissues [30], the parafoveal capillary network [31], and A-line from OCT images of arteries [32].

In this study we examine an application of quantifying three parameters (the fractal dimension, vessel length fraction, and vessel area density) from small areas of angiography images. We proposed to demonstrate a box counting method for calculating the FD of images of different sizes. Also, we developed a segmentation algorithm that allows the extraction of the vessels from the background image. We also assess the repeatability for obtaining OMAG images using an *in vivo *mouse ear model. Finally, we apply the method for quantifying small areas within an OMAG image.

## 2. Material and Methods

### 2.1. Optical Microangiography (OMAG) System

**Figure 1:**
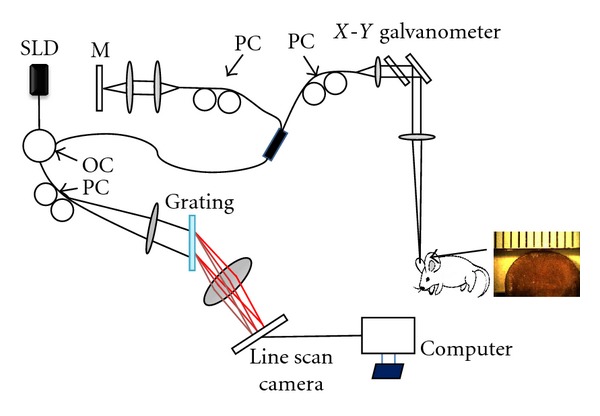
Experimental setup of the spectral domain optical coherence tomography system. SLD: superluminescent diode, OC: optical circulator, PC: polarization controller, M: mirror.

A spectral domain optical coherence tomography system has been developed [33] and is presented in Figure 1. The system contained a superluminescent diode (SLD) as a light source, with a central wavelength of 1310 nm and a bandwidth of 56 nm. The SLD provides a theoretical axial resolution of *∼*13 *μ*m in air. The light from the light source was divided into two paths via a 2 × 2 optical coupler. One light path was directed towards a mirror, known as the reference arm, and the other light path was directed towards the sample, known as the sample arm. In the sample arm, the light was coupled into a custom-designed optical system, containing a collimator, a pair of galvo mirrors, and an objective lens with a 30 mm focal length. The lateral resolution was determined to be *∼*12 *μ*m. The light backscattered from the sample and reflected from the reference mirror was recombined using the 2 × 2 optical coupler and then transmitted to a home-built spectrometer via an optical circulator for the detection of the spectral interference signal. The spectrometer had a spectral resolution of 0.141 nm, which provided an imaging depth of 2.2 mm into the sample. A high-speed InGaAs line scan camera (SUI, Goodrich Corp) was used in the spectrometer to capture the interferograms at a rate of 92,000 A-lines per second. The system sensitivity was determined to be 105 dB at a depth of 0.5 mm from the zero-delay line.

The scanning pattern and method for processing the acquired data were based on an optical microangiography technique (OMAG) [17], which allows the extraction of the three-dimensional microvascular images. Briefly, a saw tooth waveform was used to drive the x-scanner (for fast B scan), and a step function waveform was used to drive the y-scanner (for slow scan, that is, C-scan). For a B scan cross-sectional image, 256 A-lines were captured with *∼*8 *μ*m spatial interval between adjacent A-lines to cover a range of *∼*2 mm on the sample. The duty cycle for the rising side of the saw tooth waveform was set at *∼*80% per cycle, which provided a B-scan frame rate of *∼*280 frames per second. For the C scan, the 2 mm scan range was evenly divided into 200 steps with a 10 *μ*m spatial interval between them. In each step, five frames were captured and processed to extract one B-scan cross-sectional flow image. The system acquired 1000 frames to form each three-dimensional dataset. The total acquisition time for obtaining a 3D data set took *∼*3.5 seconds, which is suitable for *in vivo* experiments. Six datasets were sequentially acquired in different regions and stitched together to obtain a whole image that covered an area of ~3.5 × 5.5 mm. The smallest blood flow velocity that can be measured is 4 *μ*m/s [33].

The image processing method and the extraction of the *en face* maximum projection view image of the microvasculature network imaged by OMAG have previously been reported [33]. The *en face* projection view images were analyzed using a fractal dimension analysis method, and the vessel length fraction and vessel area density were quantified [24].

### 2.2. Experimental Protocol

We obtained *in vivo* images of the microvasculature from a mouse ear. The OMAG images were obtained at three consecutive days. The images covered an area of ~3.5 × 5.5 mm. All experiments were performed on a C57BL/6 male mouse approximately two months old. During the experiment, the mouse was anesthetized using 2% isoflurane (0.2 L/min O_2_, 0.8 L/min air), and the ear was depilated with a commercial human hair remover lotion.

### 2.3. Fractal Dimension, Vessel Length Fraction, and Vessel Area Density Analysis

To quantify the 2D *en face* projection view images, we used three quantitative parameters: fractal dimension (FD), vessel length fraction (VLF), and vessel area density (VAD). The method consists of segmenting the blood vessels from an OMAG image, which produces a binary black and white image. The segmentation algorithm consisted of three steps. First, a low-pass filter was used, which minimized elements that were smaller than a specific radius size. Then, a global threshold was used to set to zero all the pixels below that threshold. Finally, a local adaptive threshold was implemented to binarize the image based on the mean pixel value within a predefined window size. This is further discussed in Section 3.2.

**Figure 2:**
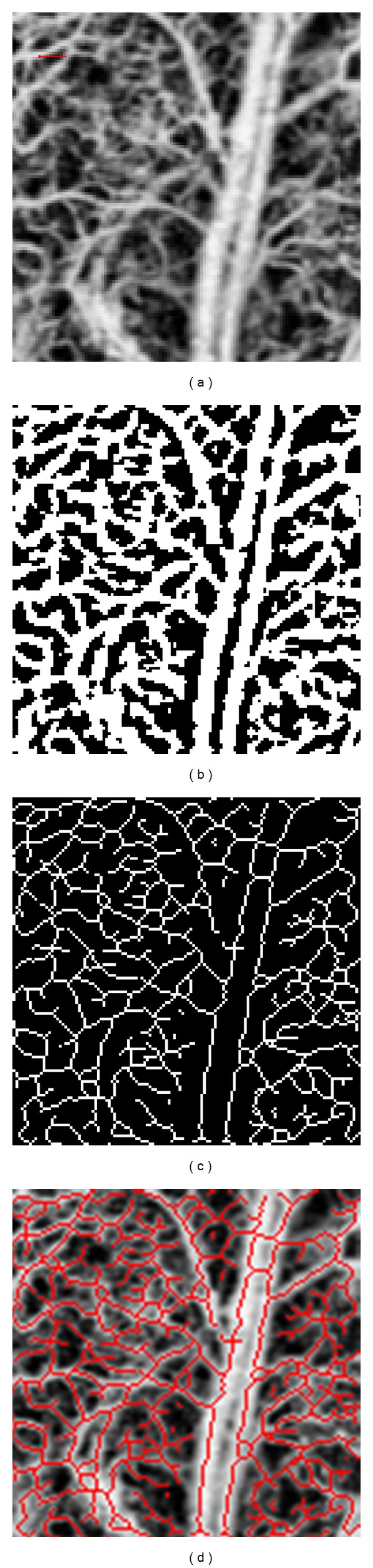
(a) OMAG image obtained from a mouse ear. Scale bar is 0.1 mm. (b) Black and white segmented image of (a). (c) Skeletonization of the segmented image (b). (d) Overlay of (c) and (a).

Figures 2(a) and 2(b) show an example of the original OMAG image (128 × 128 pixels) and its segmented counterpart, respectively. The VAD is calculated by counting the number of white pixels in the binary image, which represent the area covered by vessels, and dividing it by the total number of pixels in the image. For Figure 2(a), the total number of pixels in the image is 128 × 128 = 16,384 pixels.

The binary image is then skeletonized by reducing all the continuous white segments to a line with a single pixel width. The skeletal image is a representation of the total vessel length. The skeletonization consists of iteratively deleting the pixels in the outer boundary of the segments until a single pixel width line is obtained [34]. As a result, we obtained a collection of lines which represent the midlines of all vessel shapes. A skeletonized image is observed in Figure 2(c). The VLF is calculated by counting the number of pixels in the skeletonized image, which represents the length of all the vessels, and dividing it by the total number of pixels in the image.

Figure 2(d) shows the overlay of the original image (Figure 2(a)) with the skeleton (Figure 2(c)). It can be observed that the skeleton vessels overlap well with the vessels in the original image.

The fractal dimension was calculated over the skeletonized image using a box counting technique [35], which is a method of estimating the fractal dimension from structures that are not perfectly self-similar. Briefly, the box counting method consists of dividing a skeletonized image into square boxes of equal sizes, where the number of boxes containing a vessel segment is counted. The process is iteratively repeated with boxes of different sizes. The absolute value of the slope of the curve that shows the logarithm of the box size plotted against the number of boxes containing a vessel segment is the fractal dimension. This is further discussed in Section 3.1.

Although the FD can also be calculated over the segmented image, it has been previously demonstrated that the fractal analysis is more sensitive to changes in vascular pattern on skeletonized images than those in binary images [36]; therefore, we have opted to focus on the FD of the skeletonized images only. Both the VAD and VLF have values between 0 and 1, and the FD has a value between 0 and 2.

## 3. Results

The first part of the Results section consists on validating the method that calculates the FD and the segmentation algorithm which enables the quantification of the FD, VLF, and VAD. Then, these parameters are used to prove the repeatability for obtaining OMAG images, and a new method of studying angiography images is proposed.

### 3.1. Validation of the Fractal Dimension for Different Image Sizes

**Figure 3:**
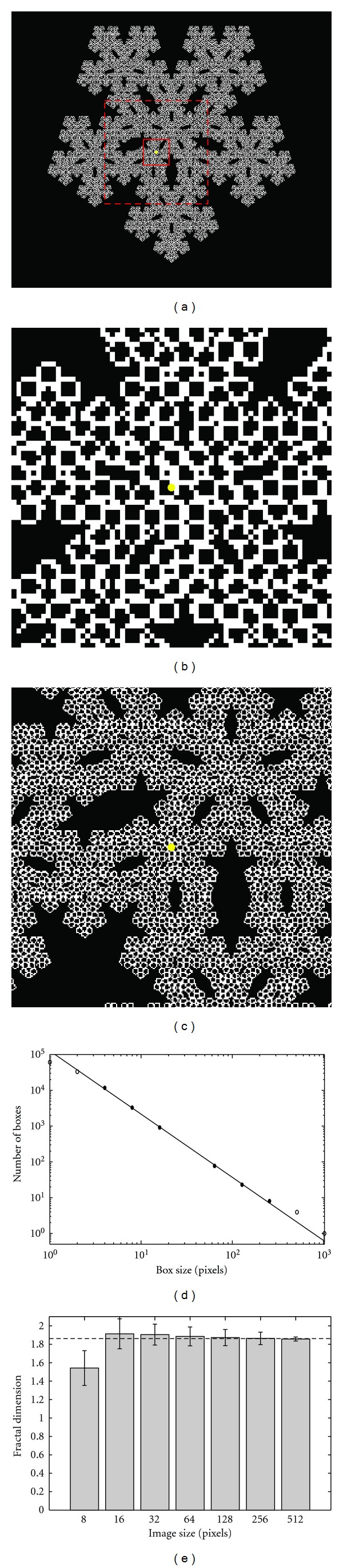
(a) Fifth iteration of a pentaflake. Cropped pentaflake for a size of (b) 64 × 64 (solid square in (a)) and (c) 256 × 256 (dashed square in (a)) pixels. (d) Box size versus the number of boxes. (e) Mean and standard deviation obtained from images of different sizes.

 To validate the accuracy of the FD calculation, an image of a pentaflake (Figure 3(a)) was used. A true fractal image can scale to infinity and has self-similarity properties at every scale. However, since we are interested in calculating the FD of an image that is limited by the pixel resolution, we opted to use as an example the fifth iteration of the pentaflake, which is a quasi-fractal image. The pentaflake has a known FD which is given by [37]

(1) $$\text{FD}=\;\frac{\log\left(6\right)}{\log\left(1+\left(1+\sqrt{5}\right)/2\right)}\approx 1.8617.$$

To calculate the fractal dimension we used a box counting method [38]. The box counting method consists of dividing a skeletonized image into square boxes of equal sizes, where the number of boxes containing a vessel segment is counted. The process is repeated several times with boxes of different sizes. The logarithm of the box size is plotted against the number of boxes containing a vessel segment. The fractal dimension is the negative of the slope of the linear part of the curve, as defined by

(2) $$\text{FD}=\;-\frac{\log_{10}\left(N\left(l\right)\right)}{\log_{10}\left(l\right)},$$

where *l* is the box length and *N*(*l*) is the number of boxes needed to cover the image. True fractal images are linear throughout the whole plot; however, quasi-fractal images are linear within a subsection of the curve.  Figure 3(d) shows an example of the logarithm of the box size versus the logarithm of the number of boxes containing a vessel segment for the whole pentaflake image observed in Figure 3(a). The circles are the data obtained from the box counting method. A straight line was fit to the linear part of the curve, indicated by the black data points.

 The calculated FD of the whole image using the box counting method is 1.845, which is very close to the value of the true fractal (1).

In the study of several microvascular phenomena, such as angiogenesis, which is the growth of new blood vessels, it is important to quantify small areas of tissue. For example, regions close to tumors may present angiogenic blood vessels (higher tortuosity and FD) compared to the healthy surrounding blood vessels [23]. Therefore, it is important to determine the smallest number of pixels within an image for which the box counting method can accurately determine the FD value.

To demonstrate the validity of using the box counting method on small images, we cropped the pentaflake image with a square window with different side lengths (8, 16, 32, 64, 128, 256, and 512 pixels). All the cropped images were centered at the same location. Figures 3(b) and 3(c) show two examples of cropped images centered at the yellow dot in Figure 3(a). Figures 3(b) and 3(c) represent image sizes of 64 × 64 and 256 × 256 pixels, respectively, which have been marked by a solid and dashed red square in Figure 3(a). Every pixel within the pentaflake image (Figure 3(a)) was selected as a center point, and cropped images of different sizes were extracted. The FD was calculated from each of the cropped images. The mean plus standard deviation of the FD for each image size is presented in Figure 3(e), where the dashed line indicates the true FD value of 1.8617. As the image size increases, the calculated FD has a better agreement with the true fractal value and the standard deviation is lower. Images with a side length of 8 pixels present a large discrepancy between the true FD value and the calculated value. Based on the box counting technique, we have demonstrated that there is a tradeoff between the accuracy of the FD value and the size of the image for which the FD is calculated; therefore, larger number of pixels in the image will have more accurate FD values.

The selection of the proper image size will depend on the application and the resolution of the system acquiring the data. The objective is to select the smallest image size which encompasses the most interesting features within an image. It is also necessary that the image size contains at least 16 × 16 pixels.

### 3.2. Validation of the Vessel Segmentation

 The method proposed requires the segmentation of the blood vessels over the angiography images. Manual segmentation is a common approach which is time consuming and is prone to error due to the experience of the grader. Therefore, the development of an automatic method for segmenting the vessels would be beneficial. An automatic segmentation algorithm would be much faster than the manual counterpart, and there would be repeatability within the same image. However, it would be difficult to separate the true vessels from the artifacts, shadows, background noise, brightness, and contrast of the different images.

We are proposing a segmentation algorithm that will enable the FD, VLF, and VAD calculation. To validate the blood vessel segmentation algorithm, we asked an expert to manually create a black and white segmentation of the image shown in Figure 2(a). The manually segmented black and white image was considered to be the reference or gold standard (GS) image. The goal was to develop a computational segmenting algorithm that will closely recreate the gold standard image.

To perform the segmentation algorithm, we first adjusted the image to increase the contrast, by making sure that 1% of the image was saturated at the low (0) and high (1) intensities. The segmentation algorithm that we developed consisted of three steps.

1. Low pass filter: this is a top hat filter which minimizes the noise in the image and eliminates elements smaller than a given radius threshold. The filter consists of erosion followed by dilation. The radius threshold is a parameter that was defined as variable 1 (V1).
2. Low global threshold: a threshold on the grayscale image was defined for which values below that threshold were set to zero and values above the threshold remained unaltered. The threshold level was defined as variable 2 (V2).
3. Local adaptive thresholding: a window size was defined for which a local threshold was determined, where the pixels below and above the threshold were set to zero and one, respectively. The threshold level was determined by the mean pixel value within the window. The window size for the adaptive threshold was defined as variable 3 (V3).

There are three variables that can be manipulated in the segmentation algorithm (V1, V2, and V3). A set of 1000 different combinations of the three variables was defined. For each combination of the variables, Figure 2(a) was segmented.

**Figure 4:**
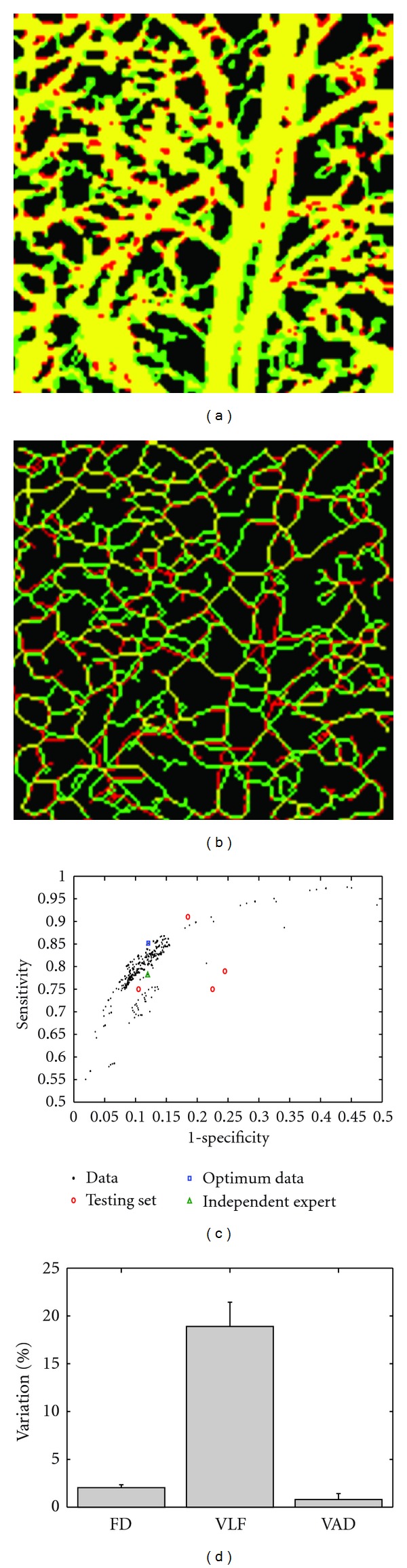
(a) Overlay of the GS image with the optimum automatically segmented image. (b) Overlay of the skeletonized GS image with the optimum automatically segmented skeleton image. (c) ROC curve of the automatically segmented image using a combination of 1000 different variables V1, V2, and V3. (d) Mean and standard deviation of the percent variation of the FD, VLF, and VAD between the manually and automatically segmented five test images.

Each computer-segmented (CS) image was overlaid with the GS image as shown in Figure 4(a). The yellow pixels represent the pixels that were selected by both the CS and the GS image, also known as true positives (TP). The green pixels were only selected by the GS image, known as false negatives (FN), while the red pixels were only selected by the CS image, known as false positives (FP). Finally, the black pixels were selected by neither image, known as true negatives (TN).

The sensitivity (Se) and specificity (Sp) from each of the 1000 overlaid images was determined by [27]

(3) $$\begin{array}{cc} \text{Se} & =\frac{\text{TP}}{\text{TP}+\text{FN}},\\ \text{Sp} & =\;\frac{\text{TN}}{\text{TN}+\text{FP}}. \end{array}$$

A receiver operating characteristic curve (ROC) was created as observed in Figure 4(c). Each of the black dots represents the Se and Sp obtained from the 1000 overlaid images. The overlaid image with the highest value of the average of the Se (85.18%) and Sp (87.91%), indicated by the blue square in Figure 4(c), corresponded to values of 15, 0.15, and 10 for V1, V2, and V3, respectively.

To validate the parameters selected for V1, V2, and V3, we obtained four new images that were manually segmented, known as the testing set. Each of the test images was computationally segmented using the optimum V1, V2 and V3, and the sensitivity and specificity have been included as red circles in Figure 4(c). Also, the same Figure 2(a) was manually segmented by an independent expert, and the Se and Sp were calculated. This value is indicated by a green triangle in Figure 4(c). As can be noted, the sensitivity and specificity are greater than 75% for all cases, indicating the validity for the method described which was used with 4 independent images and with an image segmented by an independent expert.

Finally, the FD, VLF, and VAD were calculated from the five testing set images (4 independent images and 1 independent expert). The mean plus standard deviation of the percentage variation between the values obtained by the manually and automatically segmented test images is presented in Figure 4(d). It is observed that the FD and VAD have variation of less than 5%, which indicates that those parameters are not very sensitive to the errors in the segmentation algorithm. However, the VLF presents higher-percent variations (*∼*20%) between the manual and the computational method. The differences in the skeletonized images can be observed in Figure 4(b) where several false positives and false negatives are observed.

### 3.3. Repeatability of the Optical Microangiography

**Figure 5:**
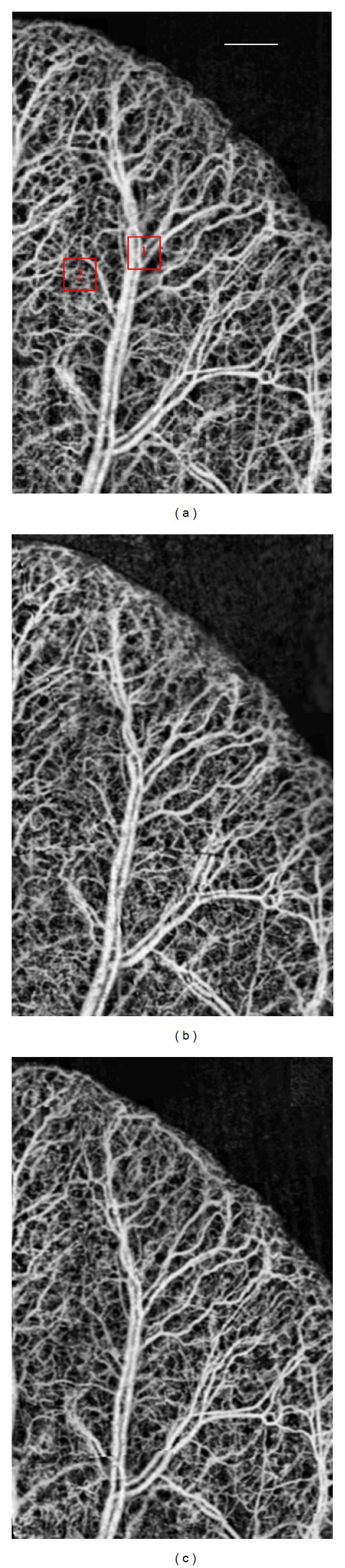
OMAG images obtained from the same mouse ear at day (a) 1, (b) 2, and (c) 3. Scale bar is 0.5 mm.

It is important to validate the repeatability for obtaining OMAG images to guarantee that the calculated values for FD, VLF, and VAD are comparable among images. Therefore, OMAG images were obtained at three consecutive days from the same region of a mouse ear. Each day, six OMAG images (~2 × 2 mm) from different regions of the mouse ear were obtained and stitched together to create a larger image (~3.5 × 5.5 mm).  Figure 5 presents the *en face* maximum projection view images from the mouse ear, which was obtained at three consecutive days. Qualitatively, it can be observed that the images appear similar among themselves.

 The FD, VLF, and VAD were calculated for the images observed in Figure 5 and presented in Table 1. The values obtained are similar among the three days indicating that stitched OMAG images are repeatable.

### 3.4. Characterizing Small Optical Microangiography Areas

**Figure 6:**
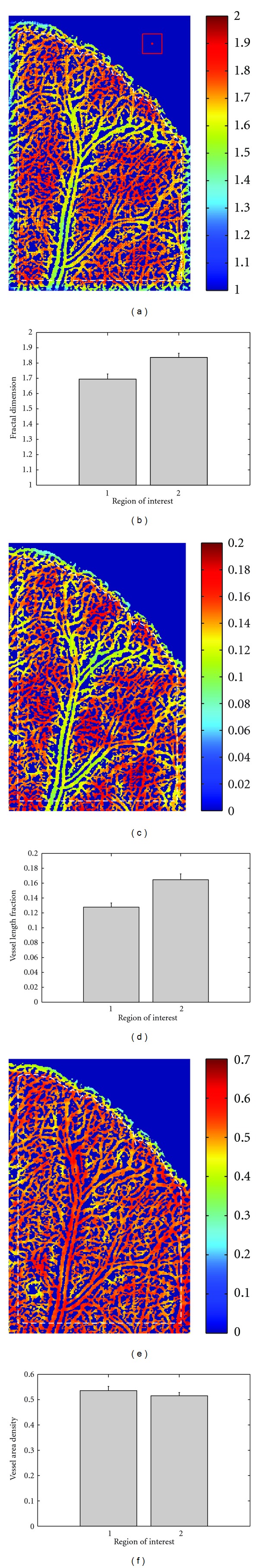
(a) Black and white segmented image multiplied by the (a) fractal dimension, (c) vessel length fraction, and (e) vessel area density of the OMAG image from day 1 (Figure 5(a)). Mean and standard deviation of the (b) fractal dimension, (d) vessel length fraction, and (f) vessel area density from the two regions of interests in Figure 5(a). The values were calculated with a 32 × 32 pixel length sliding window. The dashed lines in (a), (c), and (e) indicate the segmentation of the edges in the image.

In many applications, there is a need to quantify small microangiography regions. For example, blood vessels near and far away from a wound area may have different morphology. In the following section we propose a method to quantify small regions within OMAG images. The method consists of cropping a section of a large image, therefore, creating a smaller image. The FD, VLF, and VAD would then be calculated over the cropped image, and the values are then stored in one of the middle pixels. For example, Figure 6(a) shows a 32 × 32 pixel window (red square), and the calculated values are stored in one of the middle pixels (16,16) indicated by a red dot in the center of the red square. By sliding the window across the whole image, a color map image can be obtained. The size of the window was appropriately reduced at the borders of the image. In this application, a window size of 32 pixels (*∼*250 *μ*m) was selected, such that it was large enough to include one or more vessels within it (the largest vessels are *∼*100 *μ*m in diameter).

 The FD, VLF and VAD from the OMAG image of day 1 (Figure 5(a)) are presented in Figures 6(a), 6(c), and 6(e), respectively. For ease of visualization, the images were multiplied by the black and white segmented images. The edges of the image present an artifact due to the window having smaller size. Also the top border of the ear presents an artifact due to the window covering both the ear and outer areas of the ear. The white dashed lines in Figures 6(a), 6(c) and 6(e) demarcate the boundaries for which the artifacts are observed. Therefore, the regions outside the dashed line should be discarded.

 Two regions of interest (ROI) have been selected in Figure 5(a). Region 1 is covering large blood vessels, while Region 2 is covering small vessels and capillaries. The mean and standard deviations of the FD, VLF and VAD within the two ROIs are presented in Figures 6(b), 6(d) and 6(f), respectively.

The FD and VLF show smaller values in the regions within large blood vessels compared to the capillaries. This is expected since large vessels are fairly smooth compared to the capillaries which are more tortuous. Also, a few large vessels have a smaller length compared to several small vessels covering the same area. On the other hand, the VAD is slightly higher when it covers large blood vessels, because the area of the vessels covering the image is larger for larger vessels.

## 4. Discussion

In this study, we propose a quantitative approach for classifying OMAG images which is based on using the fractal dimension, vessel length fraction, and vessel area density. Moreover, we intend to apply these calculations on small areas within the OMAG images to obtain a color map as observed in Figure 6. The purpose is to properly characterize different regions within an image and monitor their changes, for example, compare the morphological changes in regions close and far away from an injured region, such as a burn.

The calculation of the fractal dimension is not precise given that we are not utilizing true fractal images. The images that we obtain are limited by the pixel resolution. We used a pentaflake as a reference image to validate the box counting technique. The calculation of the FD depends on the slope of the curve, which is given by (2) and observed in Figure 3(d). This method is limited by being able to properly discern the linear area of the curve. The selection of different number of data points to fit a straight line may yield different results. Images with larger number of pixels have FD values closer to their true value compared to smaller number of pixels, as shown in Figure 3(e). Therefore, it is important to acknowledge that there is a tradeoff between the image size and the accuracy of the FD value. Also, if the image size is too small (8 × 8 pixels), there is a large error in the FD calculation.

The image size used to calculate the FD must depend on the application. For this study, it was of interest to obtain a size that would include one or more vessels, such that the tortuosity could be properly estimated. An image size containing less than one vessel will not provide useful information about the vascular morphology. A large image size might provide a better FD value, but the resolution of the color map image would be reduced, therefore, providing less variation in the FD value across different areas within an OMAG image. In this study a window size was selected to incorporate at least one large vessel, as observed in Figure 6(a). Also, the system resolution was high enough that the window used had 32 × 2 pixels; therefore, the FD value is valid (Figure 3(e)).

A limitation of the method proposed is that it requires vessel segmentation over the angiography images. A manual segmentation approach may be a valid option; however, it is time consuming and is prone to error due to the difference in ability among the graders making the outcome dependent on the experience of the grader. Therefore, the development of an automatic method for segmenting the vessels would be beneficial. The advantage is that it is much faster and there is repeatability within the same image. The drawback is determining the best approach that would be universal for all images and which is capable of separating the true vessels from the noise background, such as blood vessel shadows, system noise, brightness, and contrast between the different images.

This paper describes a quantitative method for evaluating OMAG images generated from microcirculatory tissue beds *in vivo*. The parameters of the method (V1, V2, and V3) have been determined by comparing them to a manually segmented gold standard image and validated using a testing set. The testing set consisted of four new images that were manually segmented by the same expert as the gold standard image and a fifth image which was the same image as the GS but was manually segmented by an independent expert. Using a manually segmented image as a gold standard has been used in the past for other applications, such as retinal images [27]. It is important to note that the gold standard image is not perfect, given that the manually segmented images by two independent experts are not identical. In the future it would be beneficial to conduct a large-scale study with a large training and testing set and several different algorithms. However, in this study the sensitivity and specificity values obtained (>75%) are encouraging.

It is important to mention that the system resolution may affect the results. Systems with lower resolution may not be able to discern the small capillaries, while systems with higher resolution might display even smaller capillary branches. The values for V1, V2, and V3 were obtained from a gold-standard image with a given system resolution. Future studies should address how the values for FD, VLF, and VAD will vary when using systems that have different resolutions.

 The percent variations of the FD and VAD (Figure 4(d)) are less than 5%, indicating that the differences between the manually and automatically segmented images are insensitive to the calculation of these parameters. However, the VLF has a larger percent variation of *∼*20%. We attribute this discrepancy to the skeletonization algorithm, which consists of removing pixels from a continuous white shape until there is a segment with a single pixel width. If the continuous white shape (obtained by the segmentation algorithm) has one or more extra (or less) pixels, compared to the gold-standard image, the skeleton obtained may have significant differences as observed in Figure 4(b), which affect the VLF value. Although a *∼*20% variation is not a large value, this error can be reduced by improving the segmentation algorithm using a larger training and testing set as previously mentioned.

 To properly use this method, it is important to characterize the repeatability for obtaining OMAG images. OMAG images were obtained from the same region of a mouse ear at 3 different days (Figure 5), and the calculated parameters were very similar to one another (Table 1). This result is encouraging, given that one of the goals is to obtain images from the same location at different time points, and it is important to validate the repeatability in obtaining the OMAG images.

 The analysis proposed is based on using the 2D *en face* projection view images. OMAG allows obtaining 3D images of the microangiography morphology; however, the 3D images are prone to artifacts such as the shadow effect from superficial large vessels. Future studies should address the applicability of using a similar method over the three-dimensional OMAG images. This will also enable the analysis of the vessels that are located at different tissue layers.

 The method proposed has been applied specifically on OMAG images. However, it can also be applied to angiography images obtained by other methods such as photoacoustic microscopy [12].

## 5. Conclusion

In this study we have proposed to use three parameters to quantify optical microangiography images obtained using a spectral domain optical coherence tomography system. We have validated the method for calculating the fractal dimension and determined the tradeoff between image size and FD accuracy. FD values obtained from images with a large number of pixels tend to be closer to theoretical values. We have also proposed a segmentation algorithm for which three variables have been determined by studying the sensitivity and specificity of manually and automatically segmented images. The repeatability for obtaining OMAG images was demonstrated, and finally a color map of the OMAG images has been shown to demonstrate the applicability of the method for quantifying small regions within an image.

 Three parameters have been proposed to quantify angiography images. FD, VLF, and VAD provide a measure a blood vessel tortuosity, length and area, respectively. The proposed method can be used to study the angiography of biological tissues for different medical applications, such as the monitoring angiogenesis, wound healing, and capillary recruitment. It can also be used for different angiography imaging methods such as photoacoustic microscopy.

## Figures and Tables

**Table 1:** Fractal dimension, vessel length fraction, and vessel area density obtained from the images of days 1, 2, and 3 from Figure 5.

	Day 1	Day 2	Day 3
Fractal dimension	1.754	1.751	1.747
Vessel length fraction	0.12	0.12	0.123
Vessel area density	0.441	0.433	0.433
